# The coexistence region in the Van der Waals fluid and the liquid-liquid phase transitions

**DOI:** 10.3389/fchem.2022.1106599

**Published:** 2023-01-25

**Authors:** Dinh Quoc Huy Pham, Mateusz Chwastyk, Marek Cieplak

**Affiliations:** Institute of Physics, Polish Academy of Sciences, Warsaw, Poland

**Keywords:** Van der Waals fluid, phase diagam, liquid-liquid phase transitions, intrinsically disordered proteins (IDPs), molecular dynamics simulations (MD)

## Abstract

Cellular membraneless organelles are thought to be droplets formed within the two-phase region corresponding to proteinaceous systems endowed with the liquid-liquid transition. However, their metastability requires an additional constraint—they arise in a certain region of density and temperature between the spinodal and binodal lines. Here, we consider the well-studied van der Waals fluid as a test model to work out criteria to determine the location of the spinodal line for situations in which the equation of state is not known. Our molecular dynamics studies indicate that this task can be accomplished by considering the specific heat, the surface tension and characteristics of the molecular clusters, such as the number of component chains and radius of gyration.

## 1 Introduction

Cellular organelles can be either membraneless or membrane-bound. The membranes arise as droplets during a liquid-liquid phase transition (Brangwynne et al., 2009; Brangwynne et al., 2011; Shin and Brangwynne, 2017; Boeynaems et al., 2018; Elbaum-Garfinkle, 2019) as a result of thermal fluctuations. These biological droplets can be micrometers in size and exhibit hydrodynamical characteristics such as fusion (Caragine et al., 2018; Caragine and Haley, 2019). Proteinaceous liquids involved in the phase transition have been found to be composed primarily of intrinsically disordered proteins (IDPs) (Uversky, 2002; Dyson and Wright, 2005; Fink, 2005; Dunker et al., 2008; Ferreon et al., 2010; Uversky and Dunker, 2010; Babu et al., 2011; Wright and Dyson, 2015; Banani et al., 2017; Chwastyk and Cieplak, 2020; de Aquino et al., 2020) that allow for a multitude of ways to bind and aggregate.

**FIGURE 1:**
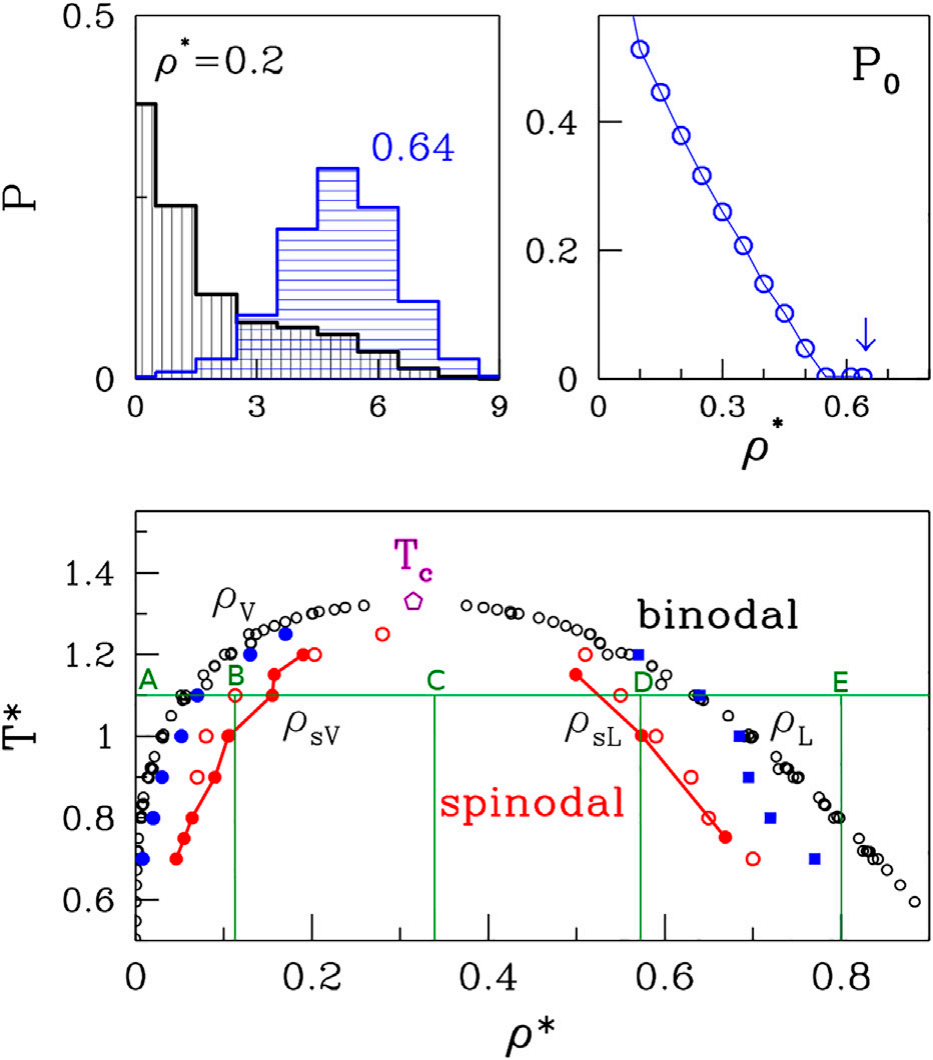
The bottom panel: The phase diagram of the van der Waals fluid. The open black and solid red circles correspond to the binodal and spinodal lines, respectively and the data originate from a summary of simulations and experiments presented by Stephan et al. (2019). The open red circles on the right of the graph represent the upper density spinodal line obtained based on the average cluster size analysis. The open red circles on the left of the graph represent low-density side and they point to the location of the left side of the spinodal line. The blue squares represent the high density binodal line based on the cubes occupancy analysis, and the blue circles represent low density binodal line. The green lines mark the densites at which the different phases are presented in Figure 5. The *T*_*C*_ marks the critical temperature. The top panels: The method of *ρ*_*L*_ determination based on the occupancy of small bins analysis.

The droplets may form only within the coexistence region of the phase diagram of the two fluids but their functionality requires that they are metastable. The paradigm model that yields such a coexistence region is the van der Waals (vdW) fluid as described by the well-known equation of state that generalizes the perfect gas law. In the density (*ρ*)—temperature (*T*) plane, the phase diagram of the vdW fluid includes the coexistence region of gas and liquid that is bounded by the inverted parabola, as shown in the bottom panel of Figure 1. Its vertex corresponds to the critical temperature (*T*_*c*_) above which one cannot distinguish between the two phases. Such a phase diagram can be obtained for the system of *n*_*m*_ monatomic particles that interact through the 6–12 Lennard-Jones (LJ) potential (Hensen and McDonald, 1973) given by:

$$\Phi_{LJ}=4\varepsilon \;\left[\;{\left(\frac{\sigma }{r}\right)}^{12}-{\left(\frac{\sigma }{r}\right)}^{6}\right],$$ (1)

where *ɛ* and *σ* are the uniform energy and length parameters. A significant increase in *ρ*, at any *T*, results in solidification. A sufficient decrease in *T* yields a similar effect. The solids may have several kinds of symmetry and the more complete schematic phase diagram can be found in ref. (Schultz and Kofke, 2018).

It should be noted that the separation into the two phases in the coexistence region can occur either through nucleation or by spinodal decomposition, depending on *ρ* and *T*. Both processes can be triggered by quenching from a temperature above *T*_*c*_, but have a different physical mechanism. Nucleation arises as a result of a rare but large energy fluctuation and is associated with metastability (Frenkel, 1955; Feder et al., 1966; Abraham, 1975; Binder and Stauffer, 1976). On the other hand, phase separation through spinodal decomposition takes place in an initially unstable system in which all fluctuations grow because there is no energy barrier (Cahn and Hilliard, 1958; Cahn and Hilliard, 1971; Langer, 1971; Huang et al., 1974; Binder et al., 1978). Thus, the generation of metastable droplets can take place only between the binodal and spinodal lines. The spinodal line for the vdW system is also an inverted parabola that is placed within the coexistence region (*cf.* The bottom panel in Figure 1). The region within the spinodal line is chaotic, unstable, and beyond a thermodynamic description. Any short-lived clusters of atoms there cannot be analogues of the “organelles.” Thus, the determination of the proper conditions for the droplet formation involves figuring out not only the position of the binodal line but also of the spinodal boundary. It should be noted that droplets of a higher (lower) density than the environment arise in the region that borders with the gas (liquid) phase. The biophysical context assumes the higher density situation.

In the absence of theoretically validated equations of state for the protein solutions, we resort to considering a simpler system: a homogeneous vdW fluid. This will allow us to test the novel concepts related to the determination of the phase diagram, giving us much-needed insight on how to deal with more complicated situations. In principle, for the vdW fluid, one can derive the free energy of the system, consistent with the equation of state, and analyze its stability. Our purpose, however, is to find alternative ways to locate the spinodal and binodal lines that could be used in molecular dynamics simulations of proteins.

## 2 The phase diagram construction

A series of simulations and experimental studies (Nicolas et al., 1979; Panagiotopoulos, 1994; Baidakov et al., 2000) of the van der Waals fluids has been reviewed by Stephan et al. (Stephan et al., 2019) and the data shown in the bottom panel of Figure 1 is based on this reference. The results are presented in reduced units (the symbols are denoted by an asterisk) that involve the length parameter, *σ*, and the depth of the energy well, *ɛ*. Density is given in units of the number of monomers per *σ* ^3^. For the cutoff value of 6.85*σ*, the critical point is at temperature *T*_*c*_ of 1.31 and density *ρ*_*c*_ = 0.316 that is consistent with a direct analysis of the equation of state.

The theoretical and experimental data (Stephan et al., 2019) were the references for our simulations. We performed molecular dynamics simulations for two systems of 4,000 particles: one for 4,000 non-bonded particles and the other one for 200 20-bead chains. During our simulations we monitored the cluster sizes, appearance of cavities and their volumes (Chwastyk et al., 2014a; Chwastyk et al., 2016), specific heat (Chwastyk et al., 2015; Chwastyk et al., 2017) as well as a number of other parameters to find a way to determine the phase diagram.

### 2.1 Details of the simulations

Our simulations were conducted by using the LAMMPS software package (Plimpton, 1995). The cut-off for LJ potential was at a distance of *r*_*c*_ = 6.85*σ*. We used the Verlet algorithm to integrate the equations of motion. The time was measured in units of $\tau_{LJ}\equiv \sqrt{m\sigma^{2}/\varepsilon }$ , where *m* is the mass of each particle. This time unit corresponds to the characteristic period of undamped oscillations at the bottom of a 6–12 potential (Chwastyk et al., 2014b; Zhao et al., 2017a; Zhao et al., 2017b). We used the integration step of Δ*t* = 0.005*τ*_*LJ*_ for the simulations of monomers and Δ*t* = 0.001*τ*_*LJ*_ for chains. The length of our simulations was 1 000 000 and 5 000 000 steps for monomers and chains, respectively, which corresponds to 5000 *τ*_*LJ*_ of total simulation time. The trajectory analysis was done based on the last 1666*τ*_*LJ*_ of the simulation in each case and the other part of the simulation was the equilibration. Two atoms were considered to be in contact if the distance between them does not exceed 1.3*σ*. Two chains are treated as belonging to the same cluster when they have at least one inter-chain contact. As a consequence, we define a cluster as a group of beads (in the case of the simulation of monomers) or chains connected by at least one contact. The chain is defined as a line of monomers connected by harmonic potential:

$$U_{\mathrm{bond}}\left(r\right)=k_{b}{\left(r-\sigma \right)}^{2},$$ (2)

where *k*_*b*_ = 75000*ɛ*/*σ* ^2^ is a force constant, strong enough to keep two atoms at distance of *σ*. This assumption was established according to the results of Kevin S. Silmore *et al.* (Silmore et al., 2017). We used the canonical ensemble (NVT) and the temperature was controlled by Nose-Hoover thermostat with damping parameter of 1.0*τ*_*LJ*_ for monomers and 10.0*τ*_*LJ*_ for chains.

### 2.2 The simulations results

**FIGURE 2:**
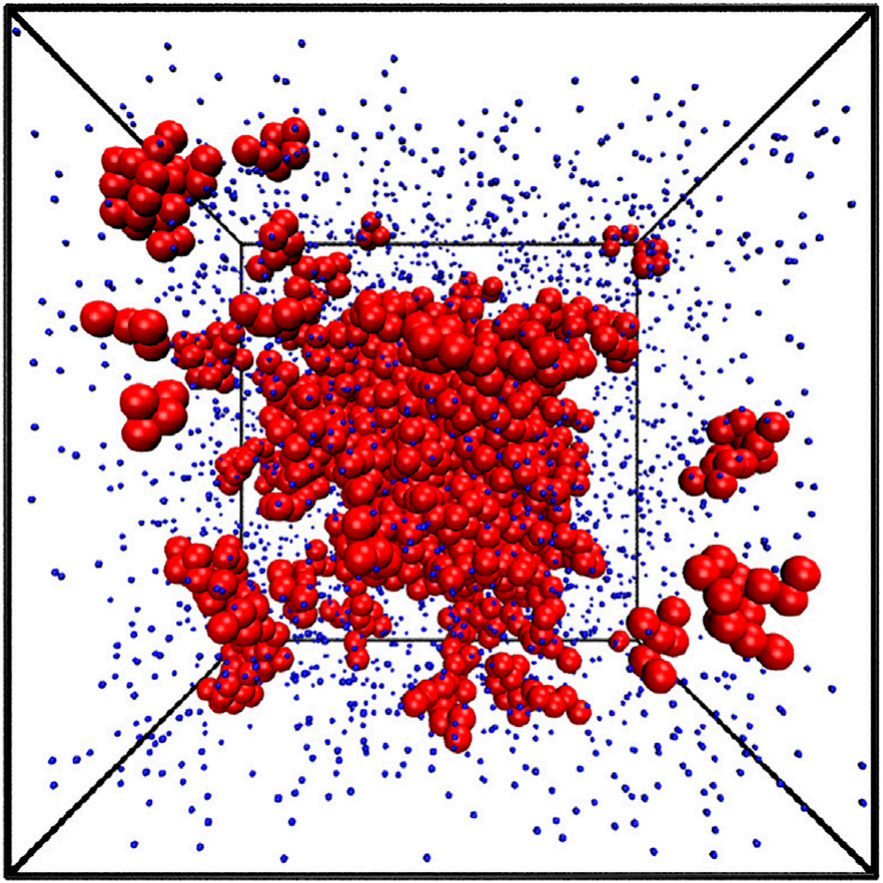
The snapshot of the equilibrium state from the simulation at ρ*=0.1 and T*=1.1. The largest cluster contains 1160 atoms and is shown at the very center. 14 smaller clusters are also highlighted. They contain between 9 and 15 atoms.

Our simulations were conducted for 90 different densities from ρ* = 0.01 to 0.90, at nine different temperatures for monomers: T* ∈ {0.6, 0.7, 0.8, 0.9, 1.0, 1.1, 1.2, 1.25, 1.3} and eight temperatures: T* ∈ {0.5, 1.0, 1.5, 2.0, 2.5, 3.0, 3.5, 4.0} for chains. The example atomic configuration is presented in Figure 2 for ρ* = 0.1 and T* = 1.1. The largest cluster composed of 1160 atoms is positioned in the center and 14 smaller clusters are highlighted by red larger balls. They contain from 5 to 15 atoms.

The top panels of Figure 1 pertain to our method of determination of the high-density branch, *ρ*_*L*_, of the binodal line. The idea is to divide the volume into small cubes with a side of order of the size of the molecule. For polymers or proteins, the radius of gyration, *r*_*g*_ would be an appropriate length scale. For monatomic molecules 2*σ* was found to be optimal. The top left panel shows the probability distributions of the cubic bins to have 0, 1, 2, 3, etc. Atoms. At the low density (ρ* = 0.2) there is a substantial probability, *P*_0_, of having an empty bin. The top-right panel shows that *P*_0_ decreases with ρ* and at around 0.64 it approaches zero. We take this value as defining *ρ*_*L*_. By considering several other temperatures we get the data points indicated as blue squares. They agree fairly well with the literature results except at the very low T*s which appear to require longer averaging.

**FIGURE 3:**
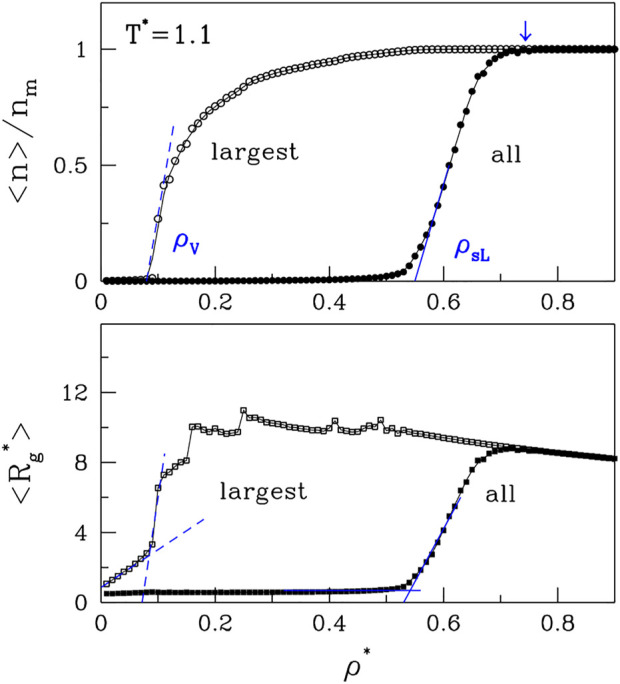
The panels, correspondingly, show the plots of ⟨*n*⟩ and ⟨*R*_*g*_⟩ vs. ρ* for T*=1.1. The data points obtained by considering all clusters are shown by the solid symbols. The open symbols correspond to the largest clusters. It would be tempting to identify the density point at which the lines ⟨*n*_*lar*_⟩ and ⟨*n*⟩ merge (the arrow at the top) as corresponding to *ρ*_*L*_. However, the location of this point practically does not depend on T*.

Let us now consider the average cluster (or droplet) sizes. These can be characterized by either the radii of gyration, *R*_*g*_, or the number of molecules, *n*, that a droplet contains. The largest possible value of *n* is *n*_*m*_. We find it useful to either consider averages over all clusters or only over the largest clusters. In the latter case, the corresponding average size will be denoted by *n*_*lar*_. The results for ⟨*n*⟩ and ⟨*n*_*lar*_⟩ are shown in the top panel of Figure 3. We observe that ⟨*n*_*lar*_⟩ undergoes a rapid growth at the low density branch of the binodal line, *ρ*_*V*_, which delineates the vapor phase. The average cluster size of all clusters also undergoes a rapid growth, but at a higher density, *ρ*_*sL*_. The growth coincides with the upper spinodal line (the open red circles in Figure 1). The lower panel shows the corresponding plots for ⟨*R*_*g*_⟩ and ⟨*R*_*g*,*lar*_⟩. They basically mimic the curves related to *n* except that the growth of *R*_*g*_ for the largest cluster is affected by the fluctuating morphology of the cluster, which affects *R*_*g*_ while not affecting *n*.

**FIGURE 4:**
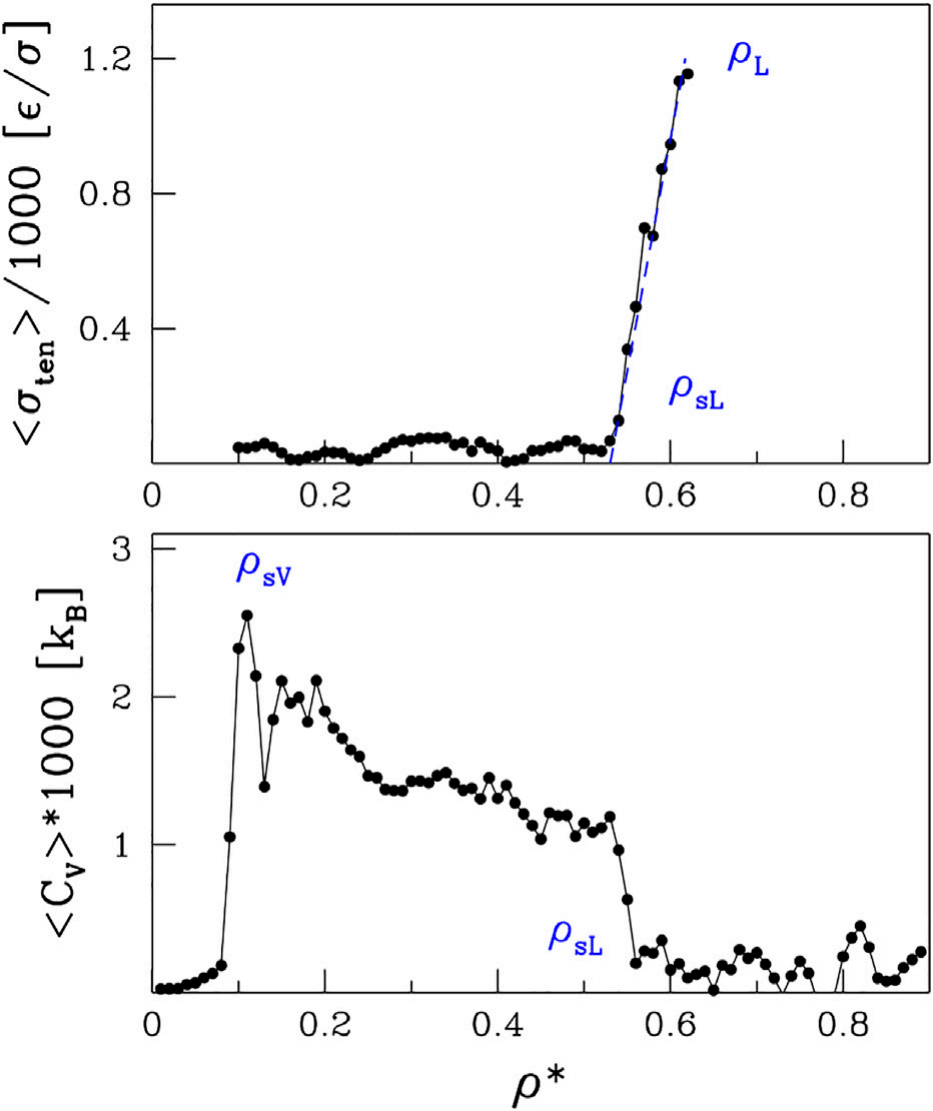
The top panel shows the coefficient of the surface tension as a function of ρ*. The lower panel shows the specific heat. Both panels are for *T* =1.1*.

In order to determine *ρ*_*sV*_, the low density branch of the spinodal line, we study the specific heat, *C*_*v*_. Since *C*_*v*_ is a measure of the energy fluctuations, we would expect volatile energy changes upon entering the non-thermodynamic spinodal region. Indeed, we observe sudden spikes in *C*_*v*_ as a function of *ρ*, as illustrated in the bottom panel of Figure 4 for T* = 1.1. The tallest of them is on the low-density side and its location is marked by open red circles on the left side of Figure 1. When going to higher densities, there is a sudden drop in *C*_*v*_ that coincides with *ρ*_*sL*_ obtained from ⟨*n*⟩.

**FIGURE 5:**
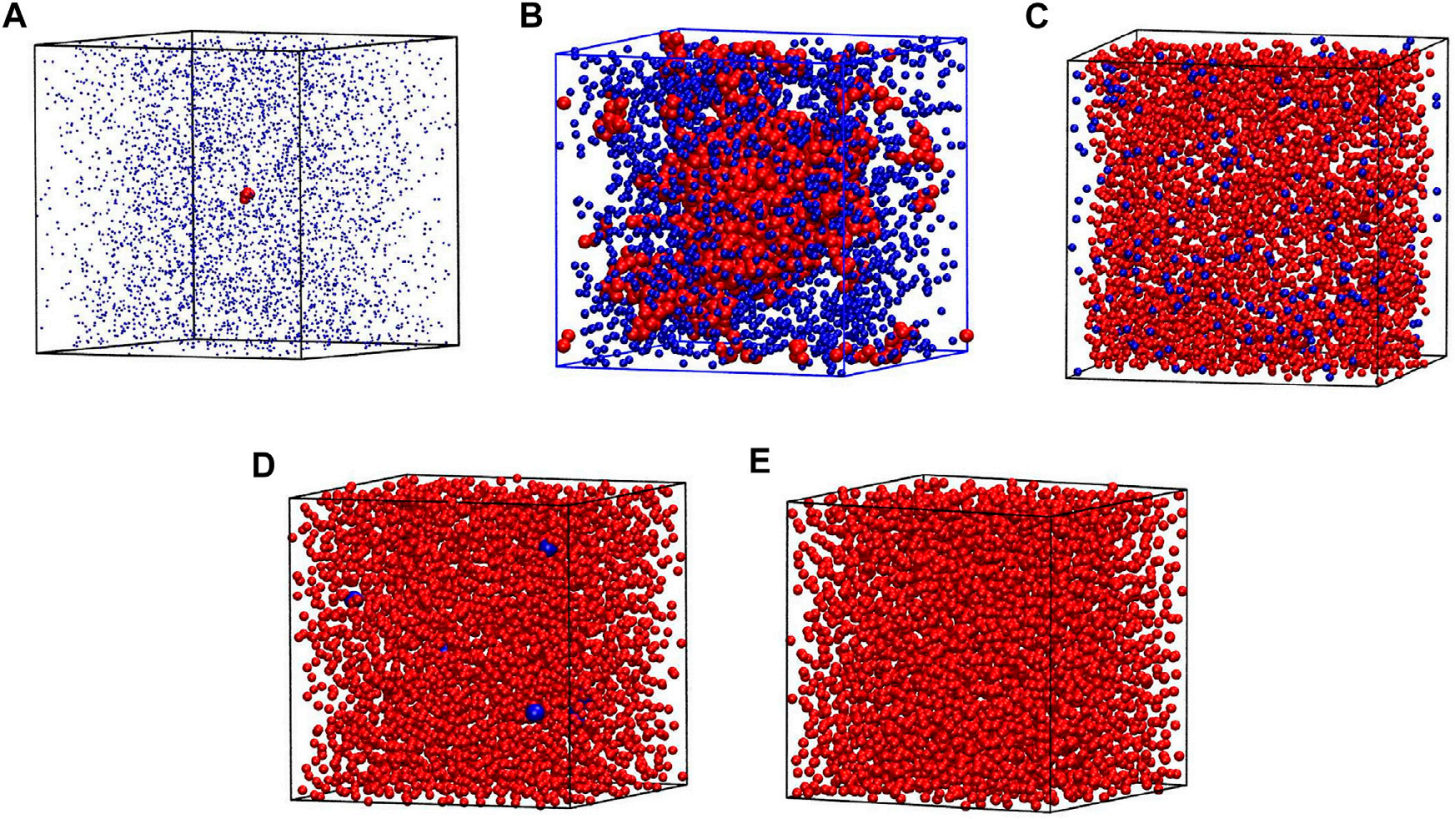
The phase separation during the density changes at T*=1.1 for system composed of 4,000 atoms. The densities of each box are ρ*=0.01, 0.11, 0.35, 0.57 and 0.8 for boxes **(A)**, **(B)**, **(C)**, **(D)**, and **(E)**, respectively. The clusters are marked by red in panels **(A,B)**. At the dense phase **(C,D)** the clusters are marked by blue. The positions on the phase diagram of particular cases are marked by green letters in Figure 1.

Yet another way to assess the boundary of the spinodal region is through the surface tension, *σ*_*ten*_. It can be derived, both theoretically and experimentally, by invoking the energy equipartition theorem (Caragine et al., 2018; Caragine and Haley, 2019) leading to *σ*_*ten*_ = *k*_*B*_ *T*/*u* ^2^ where *u* ^2^ is the fluctuation in the droplet linear size and *k*_*B*_ is the Boltzmann constant. In molecular dynamics, we take *u* ^2^ to be a fluctuation of *R*_*g*_ and average it over time. We perform this procedure for sufficiently large droplets, as they are better defined. However, to avoid the finite-size effects, we do not consider droplets that span the whole system. The surface tension, *σ*_*ten*_, calculated in this manner is shown in the upper panel of Figure 4 for T* = 1.1. The behavior of *σ*_*ten*_ as a function of density is rather irregular in the spinodal region but then there a nearly monotonic increase is observed between *ρ*_*sL*_ and *ρ*_*L*_. The phase separation induced by the density changes at T* = 1.1 is presented schematically in Figure 5. The nucleation process can be observed in panels B and D for light and dense phases, respectively. Panels A and E represent one-phase regimes.

## 3 The phase diagram for chains of monomers

**FIGURE 6:**
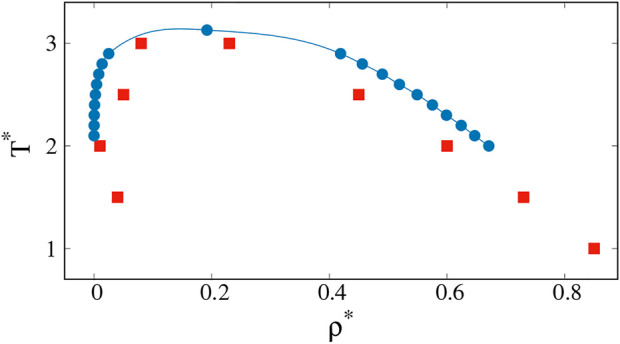
Coexistence curves (binodal lines) for chain of monomers calculated by Kevin S. Silmore et al. (2017) (blue circles) and obtained from our simulations based on specific heat analysis (red squares).

Proteins differ from the Lennard-Jones atoms discussed so far in two major ways: first, their molecules are in the form of chains, and second, the monomers in the chains are of a heterogeneous nature as they represent 20 types of amino acid. The second aspect requires special studies along the lines of Dignon et al. (Dignon et al., 2018a; Dignon et al., 2018b; Dignon et al., 2019) or Mioduszewski et al. (Mioduszewski and Cieplak, 2018; Mioduszewski and Cieplak, 2020; Mioduszewski and Cieplak, 2021) who use different coarse-grained models to analyze the protein dynamics. In addition, proteins may have inverted binodal lines when hydrophobic effects intervene (Li et al., 2002; Urry et al., 2002; Dignon et al., 2019). We now consider the first of these aspects by performing molecular dynamics simulations for *n*_*m*_ = 400 chain molecules of length 20 each. The atoms in the chains are connected at a distance of *σ*. The binodal lines for this system have been derived by Silmore et al. (Silmore et al., 2017) by using the procedure of Rowlinson and Widom (Rowlinson and Widom, 1982) in which one starts with a dense blob of molecules in the center of an elongated periodic box and reaches a heterogeneous equilibrium. These results are presented by blue points in Figure 6. The chains exhibit more cohesion, and therefore the critical point is moved up in temperature in comparison to the monomeric system.

The clusters that are analogues of the biological droplets are those that should be present immediately to the left of the left branch of the spinodal line, i.e. close to the gas phase. To the right of the right branch of the spinodal line, there are droplets of the low density regions that are essentially like cavities in the liquid phase. The cavities disappear on crossing the binodal line towards the single-component liquid phase. In numerical practice, finding the left brach of the spinodal line can be achieved by considering *C*_*v*_. This works also for the right-hand side spinodal line but monitoring the surface tension offers an additional tool. Our results for the determination of the spinodal line, based on the *C*_*v*_ analysis are presented by red squares in Figure 6.

## 4 Conclusion

In principle, a precise determination of both the binodal and spinodal line requires procedures of finite-size scaling. Our purpose here, however, was to determine quantities to accomplish the task of determining the region in which the metastable droplets could be studied theoretically. In previous theoretical studies (Dignon et al., 2018a; Dignon et al., 2018b; Mioduszewski and Cieplak, 2018; Dignon et al., 2019; Mioduszewski and Cieplak, 2020) analyzing proteinaceous droplets, no attempt was made to locate spinodal lines within the two-phase region. The proposed approach should help in such cases, as it allows for the determination of binodal and spinodal line positions for fluids of complex composition.

## Data Availability

The original contributions presented in the study are included in the article/Supplementary Material, further inquiries can be directed to the corresponding author.
